# Dynamic Tumor Immunology-on-a-Chip for Peripheral Blood-Derived Tumor-Reactive T Cell Expansion

**DOI:** 10.34133/research.0639

**Published:** 2025-03-21

**Authors:** Xin Shou, Yunru Yu, Dan Wu, Peihua Lu, Miaoqing Zhao, Yuanjin Zhao

**Affiliations:** ^1^ Department of Rheumatology and Immunology, Nanjing Drum Tower Hospital, School of Biological Science and Medical Engineering, Southeast University, Nanjing 210096, China.; ^2^ Key Laboratory of Artificial Organs and Computational Medicine in Zhejiang Province, Institute of Translational Medicine, Shulan International Medical College, Zhejiang Shuren University, Hangzhou 310015, China.; ^3^ Department of Oncology, The Affiliated Wuxi People’s Hospital of Nanjing Medical University, Wuxi People’s Hospital, Wuxi Medical Center, Nanjing Medical University, Wuxi 214023, China.; ^4^ Department of Pathology, Shandong Cancer Hospital and Institute, Shandong First Medical University and Shandong Academy of Medical Sciences, Jinan 250117, China.

## Abstract

Adoptive T cell therapy has shown great promise in the treatment of solid tumors, which, however, poses a great challenge to obtain autologous tumor-reactive T cells in a cost-effective manner. Here, we present a dynamic tumor immunology-on-a-chip, mimicking immune responses, for achieving the enrichment and expansion of tumor-reactive T cells. Tumor spheroids with uniform size can be generated by seeding tumor cells in hydrogel-embedded micropillar arrays, and could be trapped upon removal of hydrogel. Then, T cells were infused and fully contacted with these tumor spheroids under biomimetic flow conditions provided by herringbone-patterned microgrooves arrays. We found that the tamed tumor-reactive T cells could be fully activated and a rapid clonal proliferation was realized during the cultivation. In addition, these tumor-reactive T cells exhibited a specific and powerful tumor-killing capability in vitro. Thus, the suggested dynamic microfluidic chips with staged structure-transformable properties realize both the producible formation of tumor spheroids and the recapitulation of tumor-immune crosstalk to expand tumor-reactive T cells. These features indicate that the dynamic and reproducible tumor immunology-on-a-chip has potential in the preparation of therapeutic T cell products for clinical cancer immunotherapy.

## Introduction

Cancer has been a severe public disease with millions of deaths every year over the world [1,2]. Practical approaches for cancer treatment are gradually focusing on cancer immunotherapy, especially T cell-based adoptive cell therapy (ACT) [3–5]. T cells that naturally infiltrate solid tumors possess the capacity to identify tumor antigens and are promising candidates as effector cells [6–11]. Tumor-reactive T cells, a subset of T cells possessing antitumor capabilities, can be specifically expanded from tumor biopsies and have shown impressive responses in several types of cancers in clinical practice [5,12–14]. Despite many successes, challenges to obtain tumor biopsies and the long-expanded periods limit their further application in clinical trials. Therefore, effective enrichment and expansion to obtain vast numbers of tumor-reactive T cells is a critical step for preparing therapeutic T cell productions [13,15,16]. However, the current methods of in vitro expansion are simply performed by mixing tumor cells with immune effectors, which lack the reconstruction of the tumor microenvironment [17–20]. Furthermore, the insufficient killing and the unremovable tumor cells might lead to a residual risk when T cells are reinfused. Thus, in order to overcome these shortcomings, effective strategies to enrich and expand tumor-reactive T cells are still being anticipated.

**Fig. 1.**
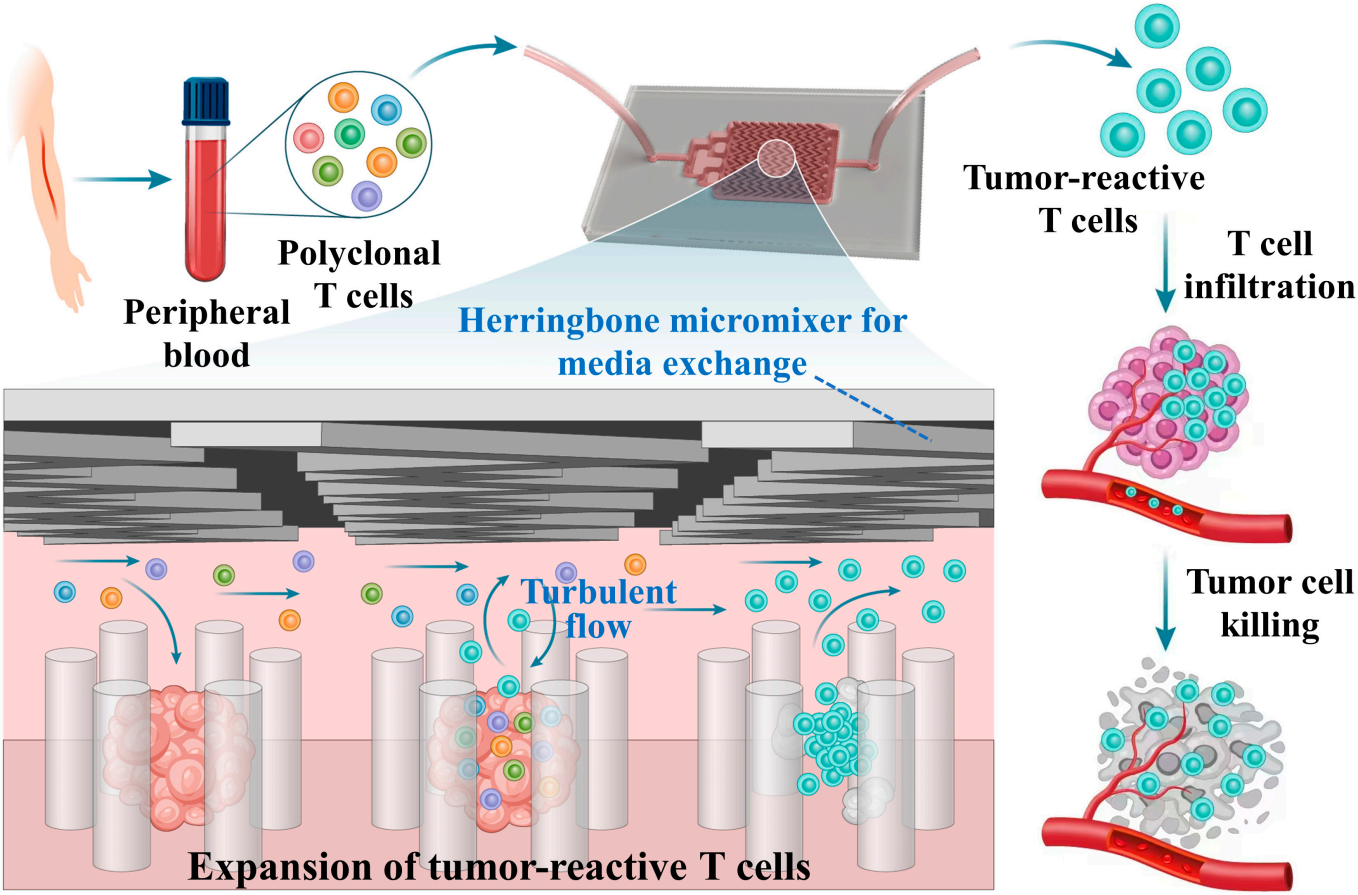
Schematic diagram illustrating the generation process of tumor-reactive T cells based on a dynamic tumor immunology-on-chip. Tumor-reactive T cells can be enriched from PBMCs and fully activated in the support of tumor spheroids, resulting in an enhanced capacity for tumor cytotoxicity.

In this paper, a novel dynamic tumor immunology-on-a-chip that mimics tumor-immune crosstalk to realize the enrichment and expansion of tumor-reactive T cells was presented, as schemed in Fig. 1. Organ-on-a-chip is a microfluidic-based culture device that simulates organ structures, physiological environments for fluid and gas exchange, and intercellular interactions [21–28]. As one typical organ-on-a-chip, tumor-on-a-chip systems are often constructed by seeding tumor cells on predesigned 2-dimensional (2D) or 3D patterns and pose advantages over high throughput, low cost, and reproducibility [29–35]. Although they have shown promising applications in cancer metastasis and preclinical drug screening, the fixed structures of these tumor-on-a-chip systems made it challenging for efficient tumor spheroid growth, dynamic tumor environment recapitulation, and in vivo blood flow dynamic simulation. In addition, to our knowledge, recent tumor-on-a-chip is seldom utilized for tumor-reactive T cell screening, and their values in enhancing T cell immunotherapy have not been fully demonstrated [36–44].

Herein, we constructed the dynamic tumor immunology-on-a-chip with enhanced tumor spheroid-immune crosstalk and efficiently expanded tumor-reactive T cells under biomimetic flow conditions (Fig. 1). The microfluidic chip was assembled by a micropillar array at the bottom layer to trap tumor cells and herringbone-patterned microgrooves at the upper layer to generate helical flow and chaotic stirring for nutrient exchange. Tumor spheroids with spherical shape and uniform size distribution were formed in alginate-based porous hydrogel arrays. After the removal of hydrogels by enzymatic digestion, the pattern changes of fluid shear stress in tumor immunology-on-chip enabled direct crosstalk between tumor spheroids and T cells for taming tumor-reactive T cells. Notably, the tumor-reactive T cells could fully be activated and rapidly proliferate, exhibiting efficient and specific tumor-killing ability in vitro. Thus, our dynamic tumor immunology-on-a-chip holds great promise in achieving efficient 3D tumor growth and high-throughput screening of tumor-reactive T cells, offering a feasible approach for autologous T cell-based personalized cancer therapy.

## Results

**Fig. 2.**
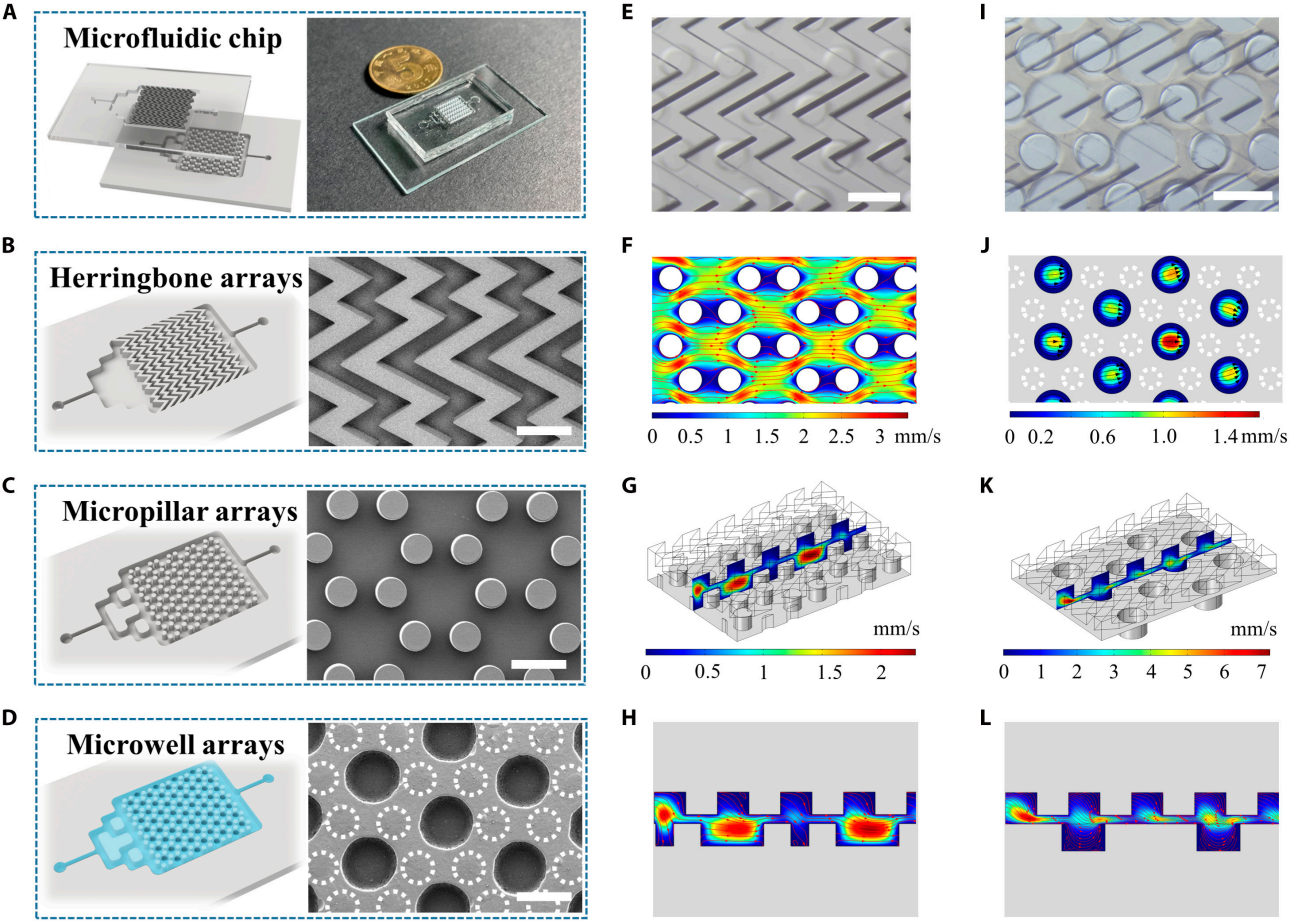
Fabrication and numerical simulation of microfluidic chips. (A) Schematics and actual image of microfluidic chip assembled with herringbone and micropillar arrays. (B) Schematics and SEM images of herringbone arrays. (C) Schematic and SEM images of micropillar arrays. (D) Schematics and SEM image of microwell arrays. (E) Optical image of herringbone-integrated microfluidic chip. (F) Flow velocity analysis of a channel in the horizontal section of (E). (G) Flow velocity analysis of a channel in a vertical cross-section of (E). (H) Fluid flow simulation along the vertical direction of (E). (I) Optical image of hydrogel-embedded microfluidic chip. (J) Flow velocity analysis of a channel in the horizontal section of (I). (K) Flow velocity analysis of a channel in a vertical cross-section of (I). (L) Fluid flow simulation along the vertical direction of (I). Scale bars, 500 μm.

In a typical experiment, microfluidic chips were fabricated by replicating the silicon mold and assembled after surface treatment with the oxygen plasma, as shown in Fig. 2A and Fig. S1. The microfluidic chip consisted of a bottom layer with micropillar arrays and an upper layer featuring herringbone-shaped microgrooves for medium mixing. The herringbone microgrooves were composed of symmetrical microchannels with an orientation angle of 60°, which were designed to generate turbulent flow, facilitate fluid transport from the herringbone to downstream edges of the microchamber, and increase the contact time between the culture medium and tumor spheres docked in the microcolumn (Fig. 2B). The micropillars were arranged in a hexagonal pattern, whose diameter, height, and separation distance were optimized to match the tumor spheroid formation needs (Fig. 2C).

To meet differentiated fluid demands in tumor spheroid culture and tumor-reactive T cell recovery during coculture, a transformable microfluidic chip was constructed. Thus far, transformable microfluidic chips were rarely reported in literatures due to the lack of environmentally responsive materials for chip fabrication. In this system, we utilize an enzymatic-degradable methacrylated alginate (AlgMA) hydrogel as a scaffold to support the formation of tumor spheroids. AlgMA was obtained by chemical modification of alginate, a natural polymer widely found in brown seaweeds. The excellent biocompatibility, rapid gelling, and hydrophilic properties of alginate would consequently benefit the application of AlgMA in tissue engineering and regenerative medicine. The results of nuclear magnetic resonance (NMR) spectroscopy showed that the methacrylate group was successfully grafted onto sodium alginate (Fig. S1A). Due to the existence of vinyl groups, AlgMA could be photopolymerized into hydrogels with the presence of initiators when exposed to ultraviolet light irradiation. In addition, AlgMA hydrogels could be further dual-crosslinked by divalent ions, including calcium and iron ions, to improve the mechanical strength. As shown in Fig. S1B, dual-crosslinked AlgMA hydrogel (AlgMA-Ca and AlgMA-Fe) possessed a higher mechanical strength compared with the single photopolymerized AlgMA hydrogel, which was attributed to the newly formed ionic bonds between guluronate residues and divalent ions. Apart from the enhanced mechanical strength, the implementation of dual-crosslinking network also strengthened the structural stability of hydrogel and mitigated its swelling. To prove this hypothesis, AlgMA hydrogels were immersed in different solutions to investigate their swelling behaviors. The results showed that the ions in phosphate-buffered saline (PBS) and Dulbecco’s modified Eagle’s medium (DMEM) culture medium markedly reduced the swelling of photopolymerized AlgMA hydrogels and prolonged their stabilization times (Fig. S2A and B), which facilitated the maintenance of designed structure.

In order to achieve the transformation of microfluidic structure, it is necessary to remove the prefilled AlgMA hydrogels without disassembling the chip device. Alginate lyase is a specific enzyme from bacteria that could oligomerize alginate. As shown in Fig. S2C, 100 U/ml of alginate lyase was able to degrade photopolymerized AlgMA hydrogels within 4 h. In contrast, calcium ion or iron ion cross-linking hindered the degradation process of alginate lyase (Fig. S2D). Besides, biocompatibility testing of alginate lyase showed that it was virtually noncytotoxic to Hepa 1-6 cells (Fig. S3A and B). Therefore, the photopolymerized AlgMA hydrogel was used to construct dynamic microfluidic chips. As shown in Fig. 2D and Fig. S4, a hydrogel-embedded microfluidic chip was successfully constructed. The hydrogel-embedded microchambers were first prepared by turning the micropillar array through specific polydimethylsiloxane molds and filled with photopolymerized AlgMA hydrogels. Apart from the spaces created by herringbone-shaped microgrooves and micropillars shown in Fig. 2E, the AlgMA hydrogel could not only completely fill the voids among micropillars but also create new microwells for tumor cell cultivation (Fig. 2I).

These delicate structures could furthermore generate turbulent flows to increase the exchange rate of culture medium in the chip. To confirm this, the distribution of fluids within the microfluidic chip was studied via numerical simulation and was analyzed through a streamline plot. It could be seen that rotational fluid distributions occurred in both the vertical and horizontal cross-section of the microfluidic channel when the flow rate of the inlet was kept at 3 ml/h (Fig. 2F to H). In detail, the highest velocity was observed in the gaps of diagonal micropillars, whereas the lowest appeared in the gaps of horizontal micropillars due to the blockage of micropillar (Fig. 2F). Contributed by the upper layer of herringbone-shaped microgrooves, turbulent flow was successfully generated in the rectangular grooves, as shown in Fig. 2G and H. Similarly, the differences in flow rates between the microwells as well as turbulent flow generated by herringbones still occurred in hydrogel-embedded microfluidic chips (Fig. 2J to L). Taken together, the designed herringbone-shaped microgrooves could generate the chaotic fluids in a microfluidic chip and improve the mixing efficiency of the culture medium, thus making it suitable for tumor spheroid culture.

**Fig. 3.**
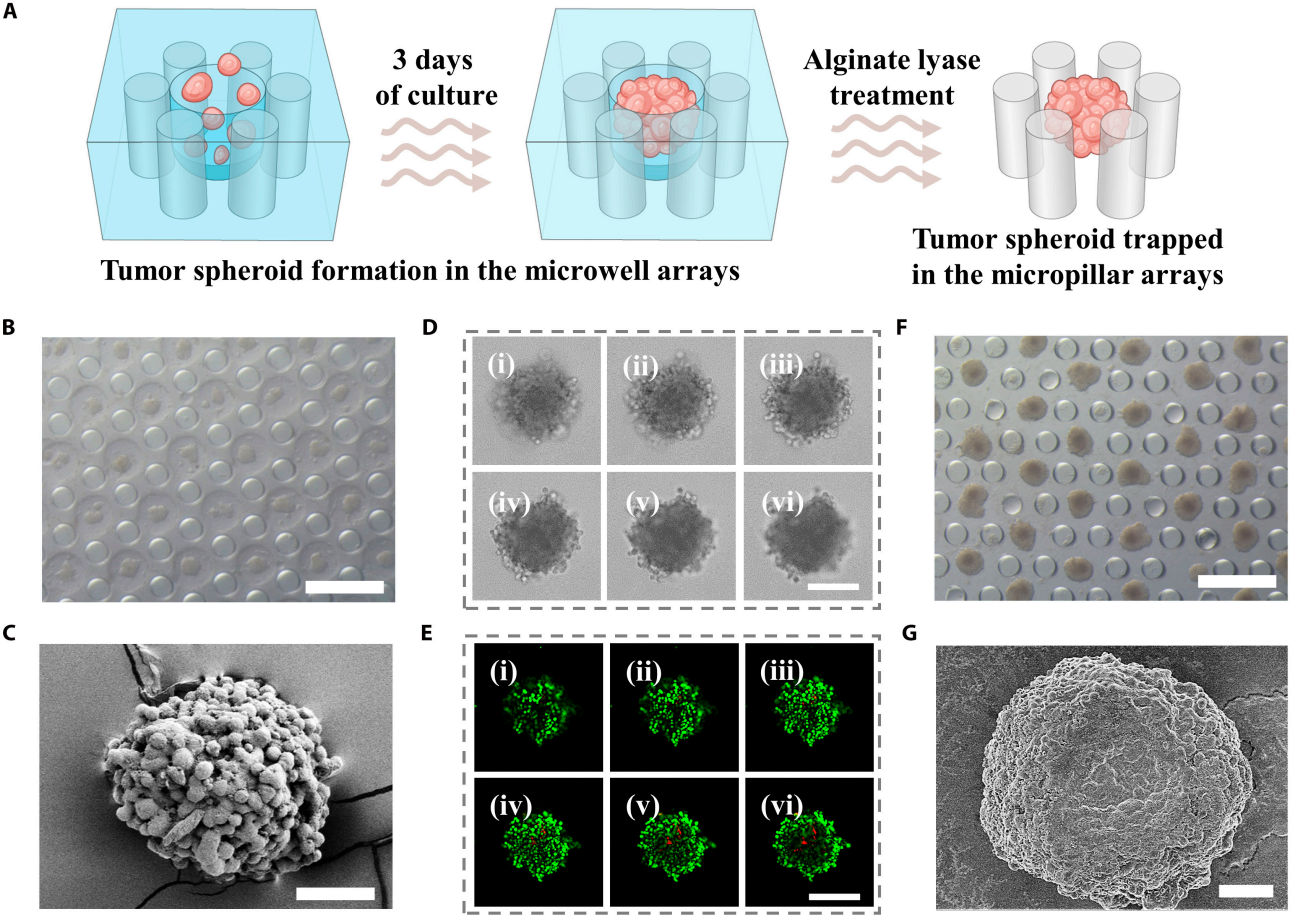
Transformability of hydrogel-embedded microfluidic chips for tumor spheroid culture. (A) Schematic diagram that depicts the formation of tumor spheroid. (B) Optical image of tumor spheroids in AlgMA hydrogel-embedded microfluidic chips. Scale bar, 150 μm. (C) SEM image of tumor spheroids prepared by microfluidic chips. Scale bar, 50 μm. (D) Optical images from top to bottom of a single tumor spheroid (i to vi) along the z-stack. Scale bar, 100 μm. (E) Fluorescence images from top to bottom of a single tumor spheroid stained with calcein-AM and PI dyes along the z-stack (i to vi). Scale bar, 100 μm. (F) Optical image of tumor spheroids in hydrogel-depleted microfluidic chips. Scale bar, 150 μm. (G) SEM image of tumor spheroid after alginate lyase treatment. Scale bar, 50 μm.

Based on the improved flow patterns resulting from the designed hydrogel architecture, the transformable hydrogel-embedded microfluidic chip was used to produce tumor spheroids. As shown in Fig. 3A and Fig. S5A, tumor cells clustered at the bottom at day 1 and became spheroid after 3 d. In order to reproducibly generate tumor spheroids, an examination was conducted to assess the relationship between the quantity of tumor cells and the resultant diameter of tumor spheroids. It was found that the diameter of the tumor spheroid increased with the seeding number of tumor cells (Fig. S5A and B). In addition, the proliferation of tumor spheroid was monitored and analyzed using cell counting kit-8 (CCK-8) assay. As shown in Fig. S5C, tumor spheroids exhibited continuous growth and revealed good cell viability. Furthermore, tumor spheroid arrays with similar size and a relatively round shape were yielded in hydrogel-embedded microfluidic chips (Fig. 3B). A tight compaction status among tumor cells in spheroid was presented in the images obtained by microscope and scanning electron microscope (SEM) (Fig. 3C and D). To investigate the ratio and location of dead cells, tumor spheroids were stained with calcein-AM and propidium iodide (PI) dyes. As shown in Fig. 3E, a slight apoptosis was found with several dead cells in the center region of the spheroid, proving the biocompatibility of AlgMA hydrogel, the appropriate structure for the constraint of tumor spheroid, and the interaction with medium flows. Further, to improve the contact efficiency between tumor cells and T cells, AlgMA hydrogel was removed after tumor spheroid formation. It could be seen that the treatment of alginate lyase could sufficiently degrade the hydrogel while keeping tumor spheroids docked between the micropillar arrays (Fig. 3F). Specifically, benefitting from the quick and mild enzymatic reaction, the structural integrity of the tumor spheroid could be maintained for further coculture (Fig. 3G and Fig. S6). Before using tumor spheroids to expand tumor-reactive T cells, tumor cells should be inactivated to avoid the immunosuppression on T cells. Therefore, we irradiated the tumor cells and tumor spheroids with 0-, 10-, 20-, 40-, and 80-Gy x-ray doses through an x-ray irradiator. To ensure the growth inability of tumor cells and select an appropriated x-ray dose, a proliferation evaluation of post-irradiative cells was carried out after 1, 3, 5, and 7 d using CCK-8 assays. It showed that the counts of irradiated tumor cells were markedly decreased compared to nonirradiated cells during the observation period (Fig. S7A and B). Furthermore, such decrease was demonstrated in a dosage-dependent manner. As a result, to achieve the most sufficient inactivation, an x-ray dose of 80 Gy was chosen to inactivate tumor spheroids (Fig. S7C). Apart from the inactivated tumor cells as feeder cells, the tumor immune microenvironment (TME) influenced tumor progression and determined the therapeutic outcome of immunotherapy. Therefore, the expression level of PD-L1 on Hepa 1-6 cells or spheroids was analyzed. We found that PD-L1 had a base-level expression on Hepa 1-6 cells or spheroids, whereas interferon-γ (IFN-γ) treatment dramatically enhanced the PD-L1 expression even on Hepa 1-6 spheroids (Fig. S8).

**Fig. 4.**
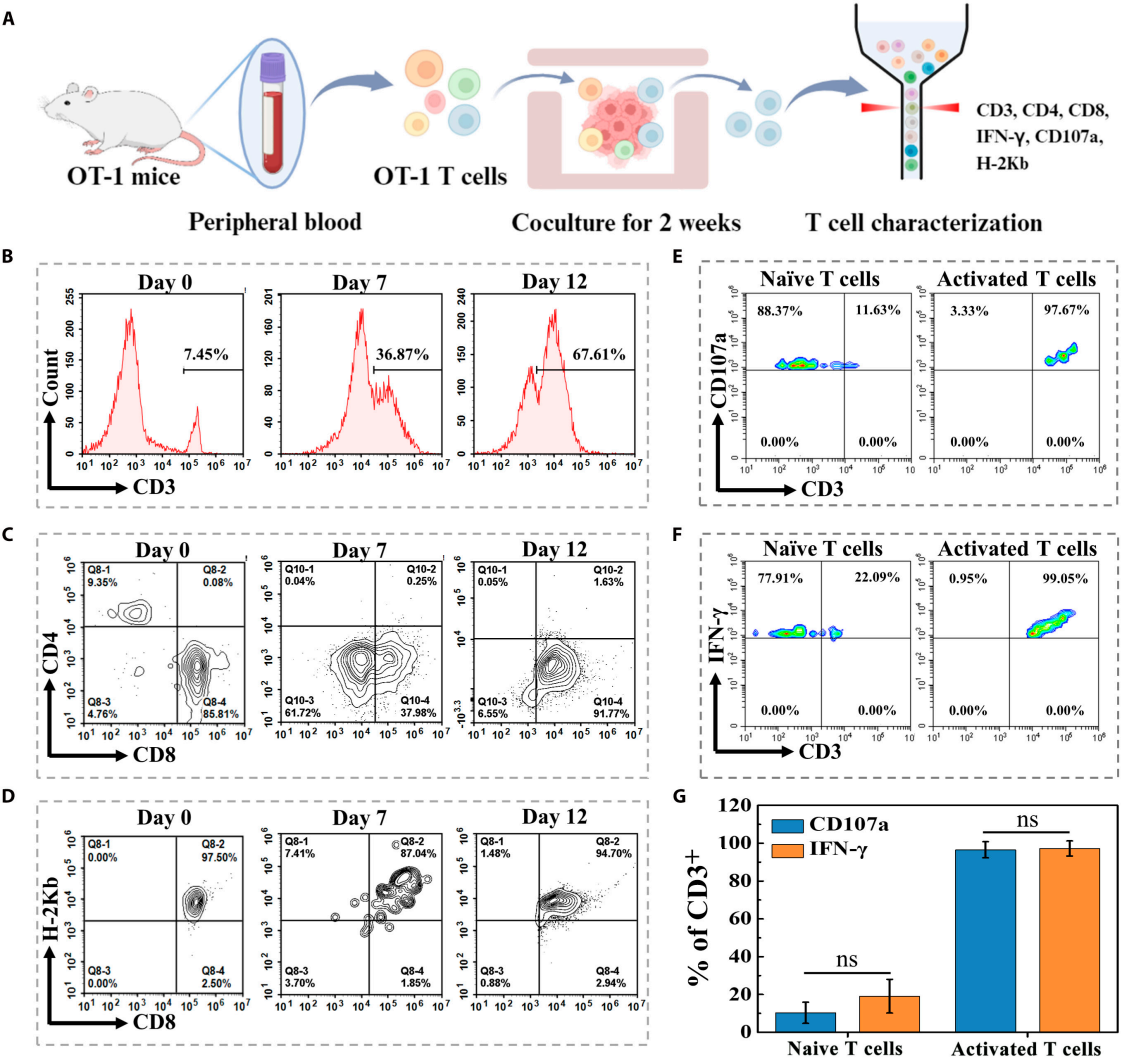
Phenotype analysis of OVA-specific T cells activated by tumor spheroids. (A) Schematic diagram illustrating the process of OVA-specific T cell expansion. The illustration was generated using BioRender under a licensed subscription (agreement number: DT27W5QUVC). (B) Purity of OVA-specific T cells before and after coculturing with tumor spheroids. (C to F) Subpopulation analysis of T cells before and after coculturing with tumor spheroids by flow cytometry. Different antibody panels targeting (C) CD4-CD8, (D) CD8-H2Kb, (E) CD3-CD107a, and (F) CD3-IFN-γ were used to characterize T cells. (G) Relative CD107a or IFN-γ expression on tumor spheroid-activated T cells in (E) and (F). ns, no significance.

Research has reported the presence of lymphocytes with tumor-reactive ability in the peripheral blood of tumor-bearing patients [45–47]. These tumor-reactive lymphocytes could be prepared by coculturing peripheral blood lymphocytes with tumor cells or tumor-derived organoids. CD8^+^ cytotoxic T cells derived from OT-1 mice have been demonstrated to effectively eradicate tumor cells expressing the peptide from chicken ovalbumin-derived peptide (OVA), both in vitro and in vivo. Therefore, we isolated lymphocytes containing anti-OVA T cell receptor (TCR) from the peripheral blood of OT-1 mice (Fig. 4A). As shown in Fig. 4B, the analysis of T lymphocyte subpopulation was conducted utilizing flow cytometry, and the percentage of CD3^+^ T cells in OT-1 mice was similar to that in nontransgenic C57 mice, whereas the transgenic cells were strongly skewed toward the CD8^+^ subpopulation (Fig. S9), aligned with results in previous studies [48]. To ensure such responsiveness, T cells from peripheral blood were activated by OVA-overexpressing Hepa 1-6 spheroids, and the proliferation of T cells was assessed (Fig. 4B and C and Fig. S10). The results showed that OT-1 T cells proliferated well and responded strongly to the tumor spheroids. Moreover, the specific recognition between anti-OVA TCR and OVA peptide antigen was further confirmed by the H-2Kb antibody (Fig. 4D). After stimulation with tumor spheroids, degranulation markers of activated T cells, such as CD107a and IFN-γ, were dramatically increased, suggesting that T cells were fully activated for cytotoxic degranulation (Fig. 4E to G).

**Fig. 5.**
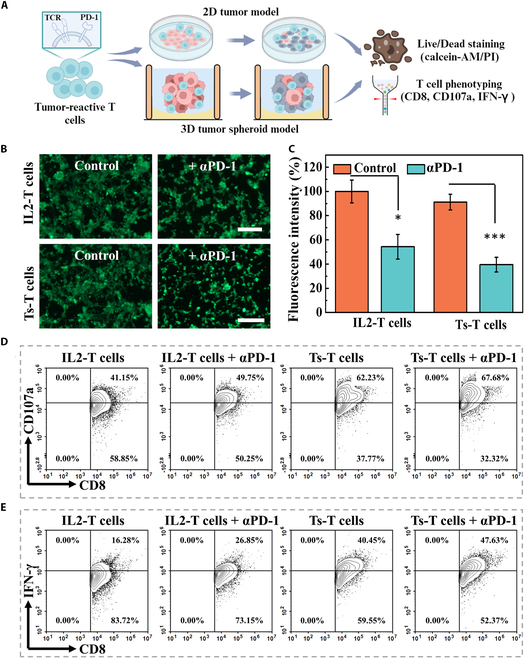
Antitumor ability of OVA-specific T cells. (A) Schematic diagram illustrating the cytotoxic effects of T cells on tumors. The illustration was generated using BioRender under a licensed subscription (agreement number: MA27W5QNLC). (B) The viability of OVA-overexpressing Hepa 1-6 cells was assessed by calcein-AM probe staining and observed by green fluorescence. Scale bar, 200 μm. (C) Statistical analysis of viable Hepa 1-6 cells in (B). (D and E) CD107a and IFN-γ expression on T cells that were stimulated by IL-2 alone or tumor spheroids (Ts).

In order to assess the cytotoxicity of enriched T cells, OVA-reactive T cells were collected from microfluidic chips and subsequently cocultured with OVA-overexpressing tumor cells (Hepa 1-6) at an effector-to-target (E:T) ratio of 10:1 (Fig. 5A). T cells activated solely by interleukin-2 (IL-2) served as the control group (referred to as IL2-T cells). Following a 24-h incubation with T cells, survival of Hepa 1-6 cells was examined by calcein-AM staining. Fluorescence images as well as the analysis on fluorescence intensity demonstrated the substantial decrease in cell viability with PD-1 blocking antibody treatment (Fig. 5B and C). In addition, the expression of CD107a and IFN-γ was markedly higher in tumor spheroid-activated T cells than in the IL-2-activated T cell group (Fig. 5D and E). These results demonstrated that OVA-specific T cells could achieve complete activation through direct interaction with tumor spheroids, enhancing the tumor cell elimination efficiency.

**Fig. 6.**
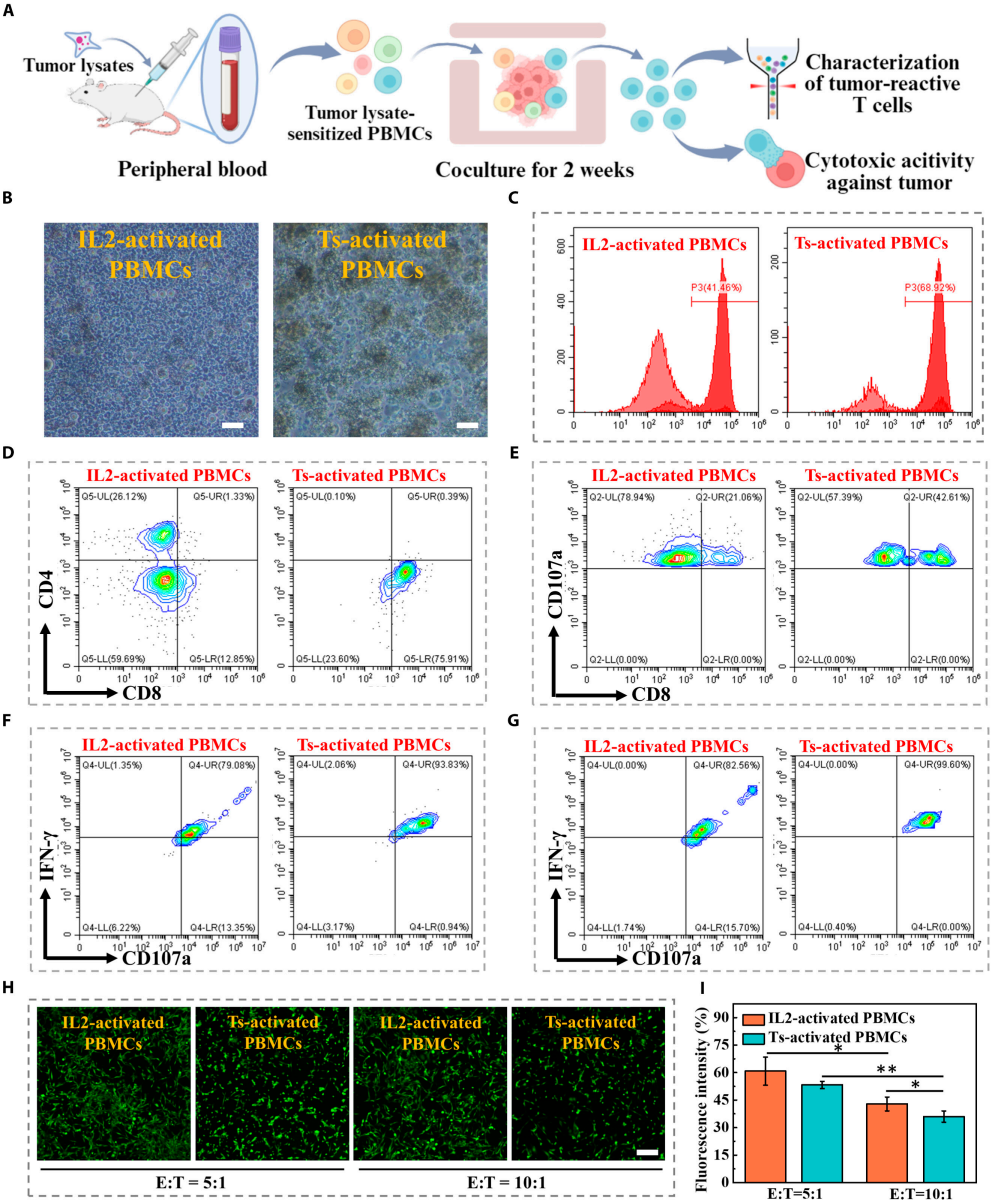
Antitumor ability of tumor spheroid (Ts)-activated PBMCs. (A) Schematic diagram illustrating the process of tumor-reactive T cell expansion and their capacity to eliminate tumor cells. The illustration was generated using BioRender under a licensed subscription (agreement number: YI27W5R4WK). (B) Bright-field images of PBMCs activated by IL-2 alone or Hepa 1-6 tumor spheroids. Scale bar, 100 μm. (C) Purity of T cells. PBMCs were activated by IL-2 alone or Hepa 1-6 tumor spheroids. (D and E) Subpopulation analysis of T cells. PBMCs were activated by IL-2 alone or Hepa 1-6 tumor spheroids through flow cytometry. T cells were stained with fluorescent dye labeling mAbs against CD3, CD4, CD8, and CD107a. (F and G) Frequencies of CD107a^+^ IFN-γ^+^ double-positive T cells analyzed from CD8^+^ (F) and CD4^+^ (G) T cell subsets. (H) Survival of Hepa 1-6 cells attacked by T cells at different E:T ratios. Tumor cells were stained using calcein-AM dye and photographed under a fluorescence microscope. Scale bar, 400 μm. (I) Viability analysis of Hepa 1-6 cells in (H).

To generate autologous tumor-reactive T cells and explore their antitumor activity, C57 mice were injected with Hepa 1-6 tumor cell lysate, and polyclonal T lymphocytes were isolated from mouse peripheral blood (Fig. 6A). After 2 weeks of coculture, tumor spheroid-activated T cells formed numbers of clusters and a dramatically increased percentage of CD3^+^ T cells were observed (Fig. 6B). The purity of enriched T cells exhibited an increase from 10% to 68% (Fig. 6C). Characterization of T cell subsets revealed that almost all tumor spheroid-activated T cells were CD8^+^, while IL-2-activated T cells were CD4^+^ (Fig. 6D). Furthermore, the degranulation of T cells was assessed in both experiment groups. It was found that T cells activated by tumor spheroids exhibited higher CD107a and IFN-γ expression than those activated by IL-2 (Fig. 6E to G). Next, the antitumor efficacy of tumor spheroid-activated peripheral blood mononuclear cells (PBMCs) was explored. Cytotoxic T cells were quantified and subsequently cocultured with Hepa 1-6 cells at varying E:T ratios for 72 h. It was found that the apoptotic number of tumor cells was positively correlated with tumor-reactive T cell numbers and a more pronounced tumor-killing activity can be observed at an E:T ratio of 10:1 (Fig. 6H and I). These results demonstrated that tumor-reactive T cells can be enriched from peripheral lymphocytes and were effective in killing autologous tumors.

## Discussion

Tumor-on-a-chip platforms serve as effective alternatives to animal models for the preclinical assessment of the therapeutic efficacy. In order to recapitulate the progress of tumor growth, angiogenesis, and metastasis, strategies including photolithography and 3D bioprinting have been devised to reproduce 1 or 2 critical components of the tumor microenvironment [49,50]. Nevertheless, owing to the intricate nature of both physiological and tumor microenvironments, current tumor-on-a-chip platforms with rigid structures still face many challenges prior to their implementation in industrial practices and clinical applications. In this study, we developed a dynamic tumor immunology-on-a-chip for tumor-reactive T cell expansion. Benefiting from the anti-adhesion properties and good biocompatibility of alginate hydrogel arrays, tumor cells seeded in the chips could successfully form uniform tumor spheroids. After removing the alginate hydrogels, the microfluidic chips exhibited a completely different structure and fluidic pattern. It was demonstrated that dynamic tumor immunology-on-a-chip, integrated with the herringbone mixer, accelerated fluid and gas exchange and effectively enhanced tumor spheroid formation.

In clinical practice, the sources of T cells used for ACT include effector T cells obtained from patients, as well as T cells that have been genetically modified to express TCRs or chimeric antigen receptors (CARs) [13,51,52]. As a subset of effector T cells able to recognize tumor antigens and exhibit antitumor capability, tumor-reactive T cells have been found in several patient’s own tissues, such as tumor-infiltrating lymphocytes (TILs) in tumor lesions, PD-1^+^ lymphocytes in peripheral blood, CD8^+^ T cells in regional lymph nodes, and CD8^+^ T cells in the spleen [16,53–55]. Despite the notable variations in clonotypes identified across different sources, tumor-reactive T cells obtained from peripheral blood remain a promising alternative source of TILs for the development of engineered cell therapies against cancer.

The success of TIL therapy trials in metastatic melanoma showed an abundance of tumor-reactive T cells existing in melanoma that can be harnessed for ACT [13]. In addition, tumor-reactive T cells are also easily found in lung cancer, ovarian cancer, pancreatic cancer, colorectal cancer, and bladder cancer [14,56–59]. Nevertheless, the proportion of tumor-reactive CD8^+^ T cells within the overall immune cell population is relatively low and varies according to the type of cancer. Consequently, there is an urgent need for effective strategies to enrich and expand tumor-reactive T cells while minimizing clonal depletion and cellular exhaustion in order to preserve their cytotoxic efficacy prior to reinfusion. In our study, tumor spheroids were used as a target for the screening of peripheral blood-derived tumor-reactive T cells. We found that tumor-reactive T cells can be fully activated in microfluidic chips with potent antitumor activity proved by tumor cytotoxicity experiments*.* These findings indicate that our dynamic tumor immunology-on-a-chip provides a valuable platform for culturing tumor spheroids and in vitro screening tumor-reactive T cells.

## Materials and Methods

### Materials

Alginate, methacrylic anhydride, PBS, and photo-initiator 2-hydroxy-2-methylpropiophenone (HMPP) were acquired from Sigma-Aldrich. Calcium chloride, sodium bicarbonate, and sodium hydroxide were acquired from Macklin. The anti-CD3ε monoclonal antibody (mAb) (clone OKT3) and the anti-CD28 mAb (clone CD28.2) were acquired from BioLegend. Mouse recombined IL-2 was acquired from PeproTech. The Live/Dead staining kit was acquired from Thermo Fisher Scientific. CCK-8 was acquired from Dojindo. The Hepa 1-6 mouse liver cancer cell line was cultured using the commercial culture medium. Fluorescein isothiocyanate-labeled anti-CD3 antibody was acquired from BD Biosciences. Phycoerythrin-labeled anti-CD56, anti-CD4, and anti-CD8 were acquired from eBioscience.

### The process of synthesizing photo-crosslinked alginate

Photo-crosslinked alginate hydrogel was synthesized by a chemical conjugation between hydroxyl groups and methacrylate groups. Briefly, alginate solution (2%, w/v) was initially prepared by dissolving alginate in H_2_O, followed by the addition of methacrylic anhydride. The grafting reaction was conducted in the dark for 24 h, during which the pH of solution was regulated over 8 using NaOH. The products were dialyzed with deionized water for 72 h followed by lyophilization for storage.

### Characterization of photo-crosslinked alginate

The structure property of alginate and AlgMA was investigated by ^1^H-NMR (QUANTUM-I-400MHz). The optical images of the herringbone-patterned microgrooves arrays and hydrogel embedded micropillar arrays were obtained by an optical microscope (CKX53, Olympus). The microstructure arrays of microfluidic chips were further analyzed by SEM (TM4000 Plus II and SU8010, Hitachi). The fluorescence image of the tumor spheroid was acquired by a stereo microscope (SZX16, Olympus) or an inverted microscope (Axio Vert.A1, Zeiss).

### Generation of tumor spheroids in hydrogel-embedded microfluidic chips

Hepa 1-6 mouse liver tumor cells were seeded at defined densities in a hydrogel-embedded microfluidic chip. Fresh medium was pumped into the chip channels, and the microfluidic chip was placed in a cell incubator with 37 °C and 5% CO_2_. After 3 d of culture, tumor spheroids were photographed and their final diameters were assessed to determine the suitable cell number initially infused.

### Swelling and degradation behavior of AlgMA hydrogels

Photopolymerized AlgMA hydrogels were prepared as described above and immersed into H_2_O, PBS, and RPMI 1640 medium. Representative images were recorded, and the swelling hydrogels were weighed to obtain the wet weight. Swelling ratio was defined as (wet weight − initial weight)/initial weight × 100%.

For enzymatic hydrogel degradation, photopolymerized AlgMA hydrogels were immersed in PBS buffer containing 0, 1, 10, and 100 U/ml alginate lyase, and the weight of AlgMA hydrogels was detected every 30 min. The degradation profile of AlgMA hydrogels was plotted at the indicated time points.

### Cytotoxicity of alginate lyase on Hepa 1-6 cells or spheroids

The cytotoxicity of alginate lyase on 2D-cultured cells or 3D-cultured spheroids was evaluated by monitoring cell proliferative activity. Cells or spheroids were mixed with a medium containing 1 U/ml alginate lyase. The assessment of cell viability was conducted using the CCK-8 assay or Live/Dead staining following 24, 48, and 72 h of culture.

### X-ray irradiation

Tumor cells (1 × 10^4^) were initially cultured in 96-well plates and subsequently exposed to different doses of x-ray irradiation, especially 0, 10, 20, 40, and 80 Gy. The dose rate of irradiation by RS-2000 x-ray irradiator was 2 Gy/min. Irradiated tumor cells were further kept in a cell incubator, and the viability was examined at predetermined time points of 1, 3, 5, and 7 d after irradiation exposure.

### Isolation and expansion of OVA antigen-specific T cells

Animal experiments were approved by the Ethics Committee of Zhejiang Shuren University with approval number: 230303. Monocytes derived from peripheral blood of OT-1 transgenic mice were separated by density gradient centrifugation. Isolated monocytes were infused into dynamic tumor immunology-on-a-chip and cultured in the medium containing IL-2 (100 IU/ml) and anti-PD-1 mAbs (200 ng/ml). Fresh medium supplemented with IL-2 was continuously pumped into a microfluidic chip throughout the culture process. After a 14-d culture period, OVA antigen-specific T cells were collected and characterized using flow cytometry.

### In vitro cytotoxicity of expanded T cells against Hepa 1-6 cells and spheroids

A total of 1 × 10^4^ Hepa 1-6 cells were initially seeded into individual wells of a 24-well plate. The next day, expanded T cells were introduced and cocultured with tumor cells at varying E:T ratios. After a 24-h incubation period, culture medium was replaced, and the viability of tumor cells was assessed using calcein-AM dye staining, followed by an evaluation with a fluorescence microscope.

To assess the antitumor activity for tumor spheroids, Hepa 1-6 cells were infused into hydrogel-embedded microfluidic chips. After 3 d of culture, Hepa 1-6 spheroids were successfully generated. Tumor spheroids were cocultured with tumor-reactive T cells, and the viability was examined by immunofluorescence staining after 24 or 72 h.

### Simulation of the fluidic flow in microfluidic chip

The numerical simulation of the fluid flow was conducted using COMSOL software. Water was selected as the model fluid to mimic the culture medium circulating within the microfluidic chip. Parameters for calculation were established with a liquid density of 998.3 kg/m^3^ and an inlet velocity of 8.33 × 10^−7^ m^3^/s.

### Statistical analysis

In this study, all results were analyzed using OriginPro 9.1 software and were presented as mean ± SD. The statistical significance among different groups was analyzed using Student’s *t* test and one-way analysis of variance (ANOVA).

## Data Availability

The data are freely available upon request.
