# Supplementary material for: Dynamic Tumor Immunology-on-a-Chip for Peripheral Blood-Derived Tumor-Reactive T Cell Expansion
Source: Research (Wash D C). 2025 Mar 21;8:0639. doi: 10.34133/research.0639 (PMC11927211; doi:10.34133/research.0639)
Supplement: Supplementary 1 — Figs. S1 to S10 [file research.0639.f1.zip › research.0639.f1.docx]

SUPPLEMENTARY MATERIALS


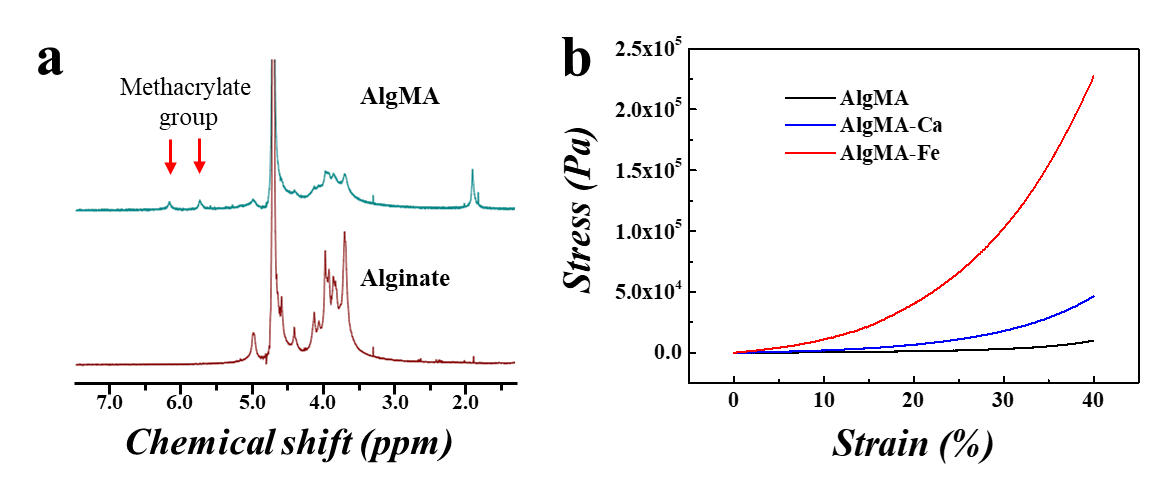


**Figure S1. Characterization of synthesized methacrylated alginate (AlgMA).** (a) Structural characterization of alginate and AlgMA detected by ^1^H-NMR spectroscopy. (b) Stress-strain curve of AlgMA hydrogel recorded by universal material testing machine.

**
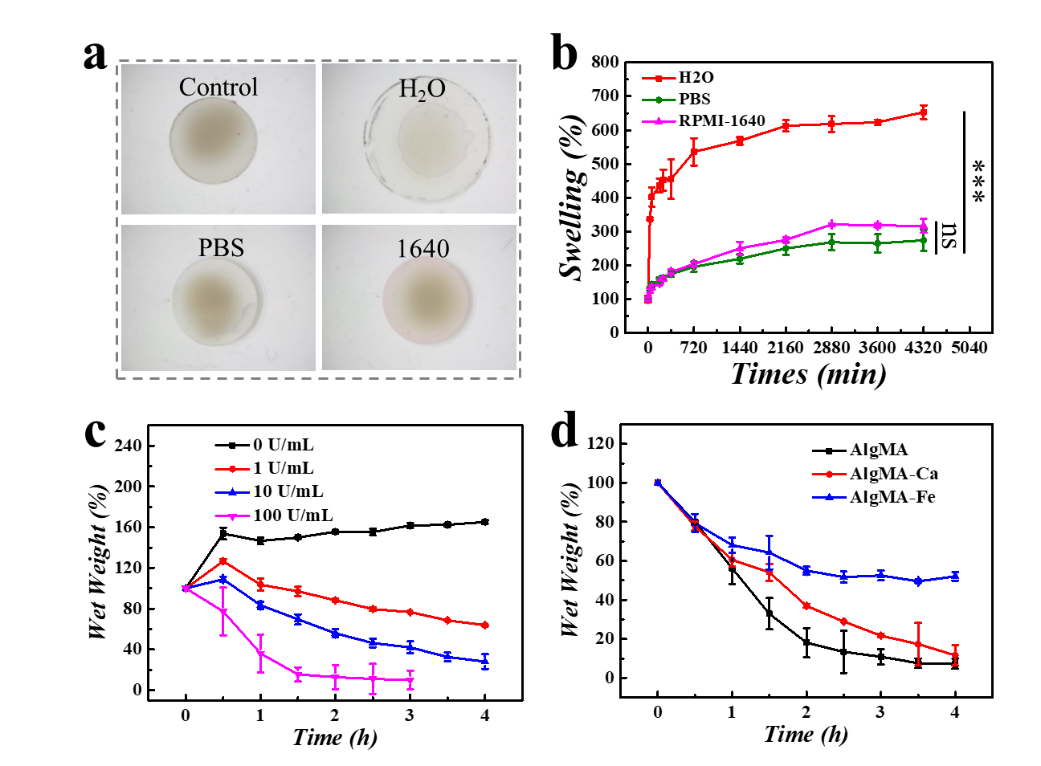
**

**Figure S2. Swelling behavior and enzymatic degradation of AlgMA hydrogel.** (a) The swelling ability of photo-crosslinked AlgMA hydrogels in different solutions. (b) The swelling ratios of AlgMA hydrogels in different solutions. (c) Degradation profile of AlgMA hydrogel treated with different concentration of alginate lyase. (d) Degradation profile of AlgMA hydrogels at the indicated timepoints.

**
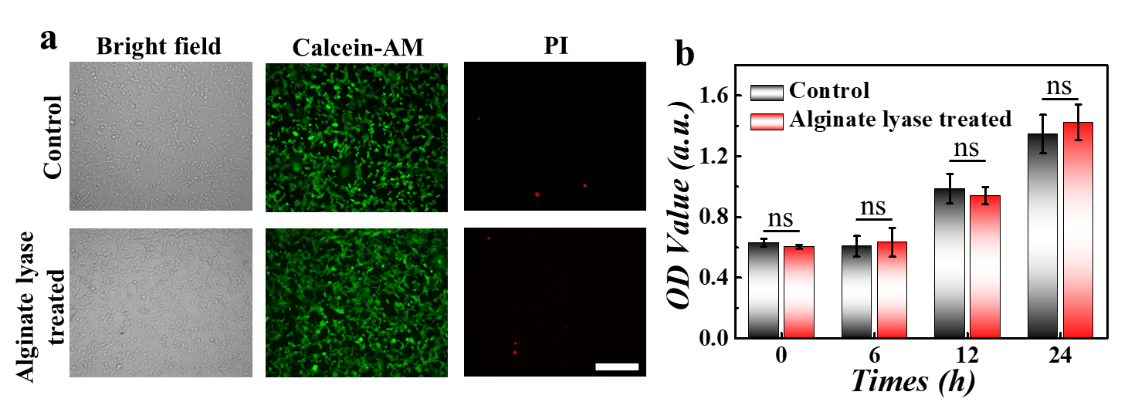
**

**Figure S3. The cytotoxicity of alginate lyase on Hepa 1-6 tumor cells.** (a) Representative live/dead staining image of 2D-cultured Hepa 1-6 cells. Hepa 1-6 cells were cultured with or without alginate lyase. Scale bar, 200 μm. (b) The viability analysis of Hepa 1-6 cells in (a). Ns, no significance.


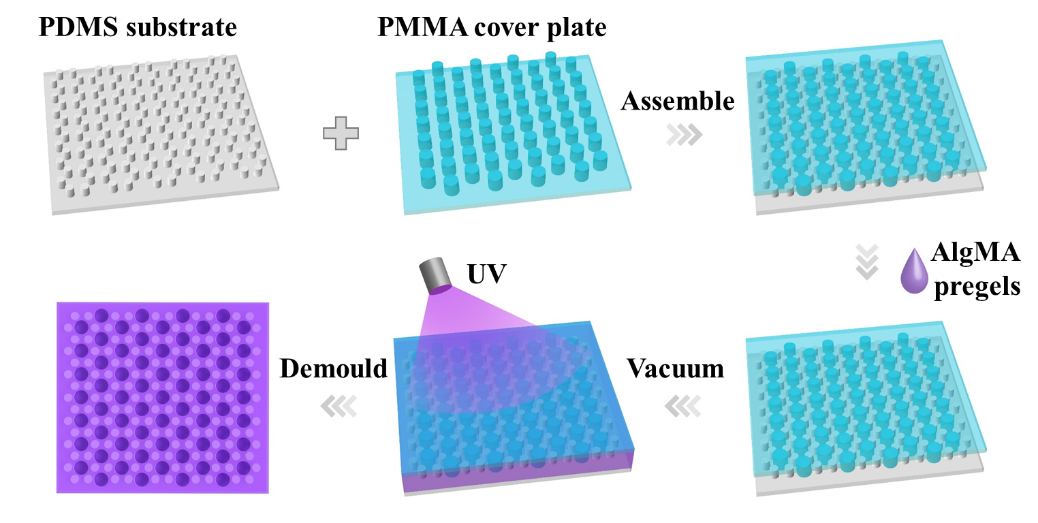


**Figure S4. Fabrication progress of hydrogel embedded micropillar arrays.**

**
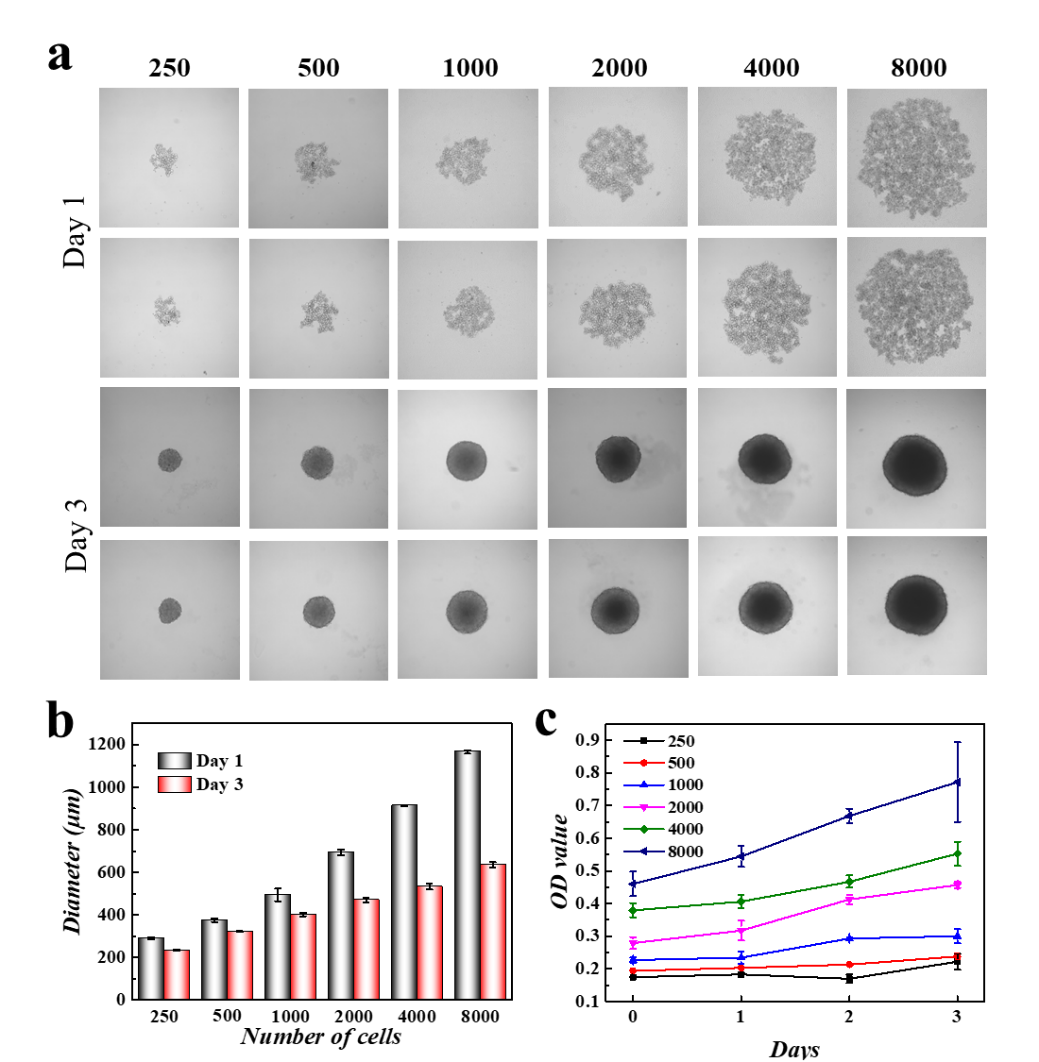
**

**Figure S5.** **Generation of Hepa 1-6 tumor spheroids.** (a) Size control of Hepa 1-6 tumor spheroids grown on microfluidic chip. (b) Diameter analysis of Hepa 1-6 tumor spheroids in (a). (c) Growth rate of Hepa 1-6 tumor spheroids grown on microfluidic chip at the indicated timepoints.


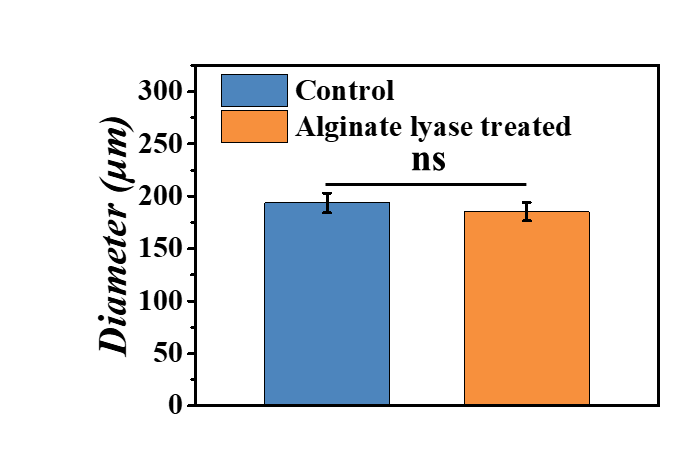


**Figure S6. The cytotoxicity of alginate lyase on Hepa 1-6 tumor spheroids.** The statistical analysis of tumor spheroid diameter. Hepa 1-6 tumor spheroids were cultured with or without alginate lyase.


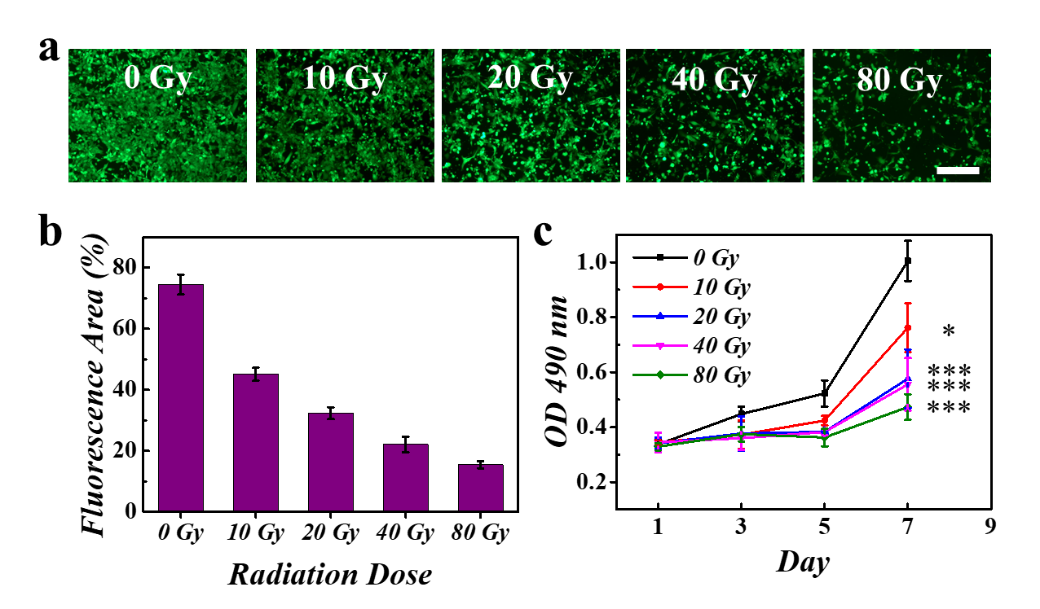


**Figure S7. X‑ray irradiation efficiently inhibited the proliferation of Hepa 1-6 cells.** Tumor cells were irradiated with different doses of X-ray (0, 10, 20, 40, 80 Gy). (a) The proliferation of non-irradiated or irradiated Hepa 1-6 cells on day 7. Scale bar, 400 μm. (b) The statistical analysis of fluorescence area in (a). (c) Cell viability analysis of irradiated Hepa 1-6 cells at predetermined time points after irradiation exposure. Ns, no significance.


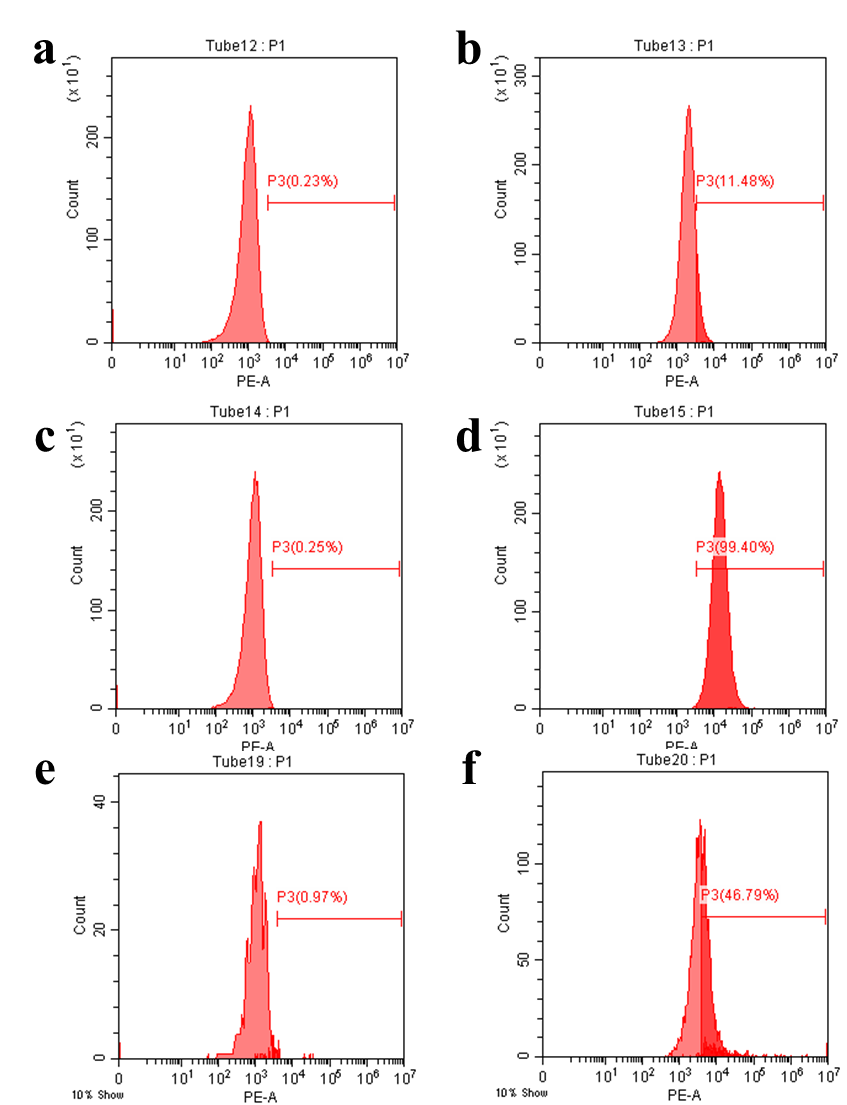


**Figure S8. PD-L1 expression analysis of Heap 1-6 cells and tumor spheroid.** (a, c, and e) Isotype control. (b) Hepa 1-6 cells stained with antibody against PD-L1. (d, f) Hepa 1-6 cells (d) or tumor spheroids (f) were treated with IFN-gamma and stained with antibody against PD-L1.


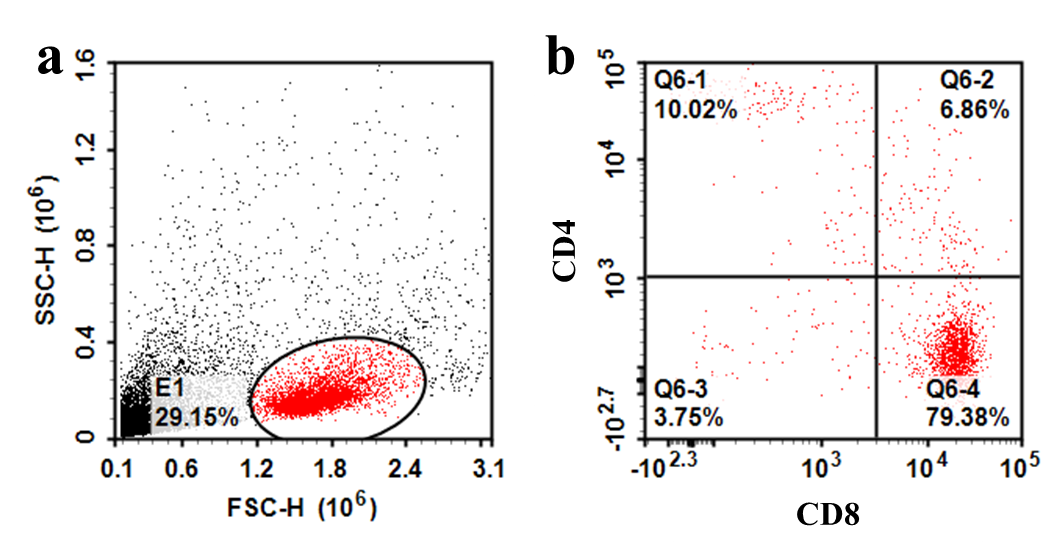


**Figure S9. Flow cytometry analysis of T cells from OT-1 mice.** (a) Gating strategy for CD3^+^ T cells. (b) CD4 and CD8 expression on CD3^+^ T cells

**
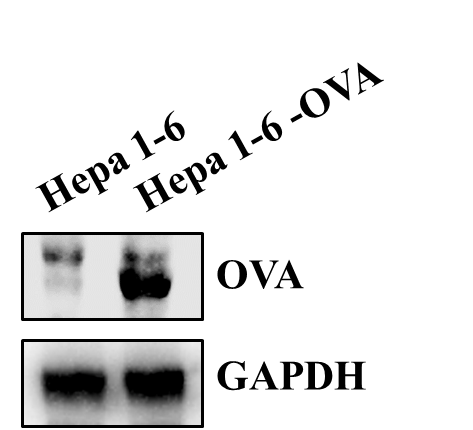
**

**Figure S10. OVA expression analysis of OVA-overexpressing Hepa 1-6 cell line based on western blotting.**
